# New discoveries on how DICER efficiently processes pre‐miRNA

**DOI:** 10.1002/mco2.430

**Published:** 2023-11-29

**Authors:** Qi Weng, Qi Wu, Quan Zheng

**Affiliations:** ^1^ Department of Pharmacy Quzhou People's Hospital The Quzhou Affiliated Hospital of Wenzhou Medical University Quzhou China; ^2^ Core Facility Quzhou People's Hospital The Quzhou Affiliated Hospital of Wenzhou Medical University Quzhou China

## Abstract

The latest study identified the “GYM motif” on precursor microRNA (pre‐miRNA) for the first time and highlighted its key role in DICER specifically recognizing and efficiently cleaving pre‐miRNA to produce miRNA. In addition, the dicing state of DICER efficiently processing pre‐miRNA was revealed, which was previously difficult to capture.

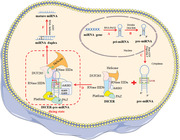

1

Two emerging studies published back‐to‐back in *Nature* elucidated the mechanism by which human DICER (hDICER) recognizes and processes precursor microRNA (pre‐miRNA), highlighted the critical function of the “GYM motif” (paired G, paired pyrimidine [Y] and mismatched C or A [M]) in pre‐miRNA processing, and also provided structural insight into pre‐miRNA processing by analyzing the cryo‐electron microscopy (cryo‐EM) structure of the hDICER–pre‐miRNA complex in the dicing state.^1^, ^2^

RNA silencing mediated by miRNA and small interfering RNA is a most important post‐transcriptional gene expression regulation involved in regulating a variety of physiological and pathological processes, including development, metabolism, aging, and tumors.^3^ Notably, DICER's precise selection of pre‐miRNA cleavage sites is critical in miRNA bio‐synthesis. In the past, it has been believed that the recognition and cleavage of DICER substrates strictly follow the mechanism of “ruler”‐like counting from the 5′ and 3′ ends to determine the cleavage site: in the role of a “molecular scale,” DICER measures 22 nt from the pre‐miRNA's terminus, where the 3′ pocket recognizes the 3′ end and 5′ pocket recognizes the 5′ end.^4^ In 2018, Wang's team reported the cryo‐EM structure of apo‐hDICER–TRBP complex and two different structural states of pre‐let‐7 combined with the hDICER–TRBP complex.^5^ This work revealed the hDICER–TRBP complex's ability to boost the stability of the pre‐miRNA's stem duplex in a pre‐dicing state, thus ensuring the precise length of the miRNA product. However, the active dicing state of hDICER in miRNA biogenesis needs to be studied in terms of structure and mechanism. In a new study, Kim's group^1^ first revealed the existence of a conserved region near the cleavage site, called the “GYM motif.” This motif is frequently prevalent in pre‐miRNAs of all metazoan species and has a significant role in the evolution of miRNAs in eumetazoan.^1^ The GYM motif facilitates site‐specific processing of pre‐miRNAs and can override the previously determined “ruler‐like” counting mechanism. During the same period, Kim's group collaborated with Roh's group to reveal the cryo‐EM structure of the hDICER–pre‐miRNA complex in a dicing state and explain how hDICER specifically selects its substrate.^2^

In order to investigate whether DICER's recognition of substrates is sequence dependent, Kim's group randomly synthesized a large number of pre‐miRNA variants and incubated them with purified hDICER proteins.^1^ Then, the pre‐miRNA variants that had been cleaved were sequenced, and the processing efficiency of different sequence characteristic variants was quantified. The authors identified a sequence factor on the pre‐miRNA that determines the cleavage site—the “GYM motif.” It is worth noting that base pairing within pre‐miRNA is generally beneficial for processing, but the mismatch of M is related to processing efficiency—GC dinucleotides enhance the impact of mismatch and improve processing efficiency. Furthermore, the authors found that the C‐terminal of double‐stranded RNA‐binding domain (dsRBD) plays a crucial role in identifying mismatches, with DICER primarily using its residues R1855 and E1859 to recognize mismatched M on the GYM motif. In conclusion, the authors revealed that the existence of a “GYM motif” on the pre‐miRNA substrate is the decisive factor for hDICER to determine the cleavage site. In addition, pre‐miRNA with a high‐score GYM motif is precisely and efficiently processed by DICER to produce miRNA, thus contributing to the efficacy of RNA interference.

Due to the lack of active structures, the structural underpinnings of hDICER's substrate specificity remain unclear. Thus, Kim's group collaborated with Roh's group to determine the cryo‐EM structure of apo‐hDICER and hDICER–pre‐miRNA^GYM^ in a dicing state.^2^ This work revealed the dynamic spatial rearrangement of various structural regions of hDICER during the catalytic state transition, and explained the process of hDICER specifically identifying its substrates (Figure 1). Through the cryo‐EM structure of hDICER–pre‐miRNA^GYM^, the authors observed that hDICER contacts pre‐miRNA extensively on the surface, and pre‐miRNA undergoes cleavage reactions at the catalytic centers formed by intramolecular dimerization of RNase III a (RIIIDa) and RNase III b (RIIIDb). By comparing the cryo‐EM structures of apo‐hDICER and hDICER–pre‐miRNA^GYM^ in the dicing state, the authors found that the DUF283 domains and N‐terminal helicase become flexible in the cleavage state. In addition, near the catalytic site of the stem on the pre‐miRNA, the authors observed that the C‐terminal of dsRBD in the dicing state moved away from the catalytic center, while the helical structure of the pre‐miRNA underwent a localized conformational distortion, which alleviated the spatial conflict between the dsRBD and the pre‐miRNA, thus allowing the pre‐miRNA to be recognized. Furthermore, the authors observed that RIIIDa and RIIIDb's α‐helices 2 and 3 generated substantial electrostatic contacts with the pre‐miRNA's upper stem region, promoting recognition of the dsRNA. Notably, the arginine residue (R1855) close to the GYM motif mismatches with C‐C to generate a hydrogen bond, demonstrating once more that R1855 of the dsRBD recognizes the mismatch of the GYM motif. More importantly, the authors found that the pre‐miRNA's 5′ and 3′ ends were fixed in the 5′ and 3′ pockets, respectively, in the dicing state. The 3′ pocket was conserved, but the pre‐miRNA's 5′ end had a special twisted conformation to accommodate a single DICER homolog with various substrates. Through mutation experiments on R821 of hDICER, the authors observed significant alterations in the cutting points of miRNA, resulting in a full loss of 5′ counting and a decrease in miRNA abundance, especially in members of the tumor‐inhibiting let‐7 family. These results indicate that 5′ pocket mutations can result in cancer and underline the significance of 5′ end identification.

**FIGURE 1:**
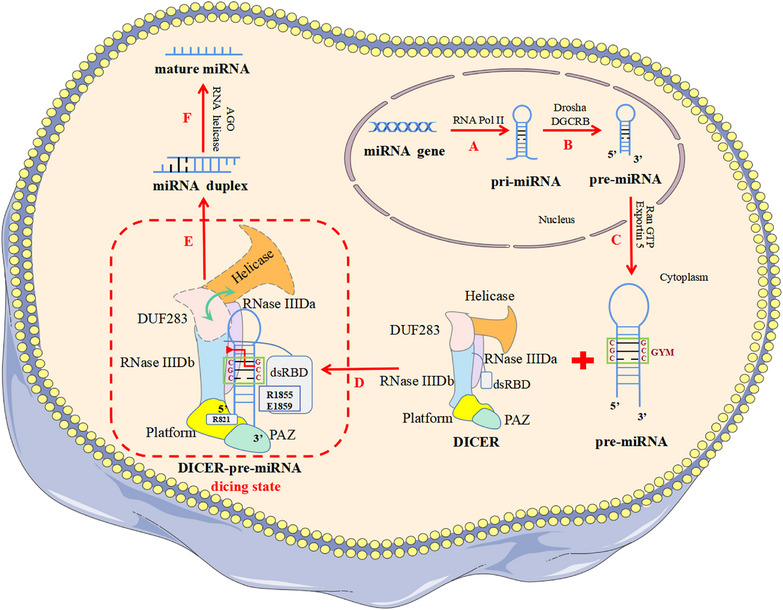
DICER specifically recognizes and efficiently processes precursor microRNA (pre‐miRNA) to produce miRNA. (A) The miRNA gene is transcribed into a primary miRNA (pri‐miRNA) by RNA polymerase II in the cell nucleus. (B) The pri‐miRNA is processed into the pre‐miRNA by a microprocessor complex composed of Drosha and DiGeorge Syndrome Critical Region 8 (DGCR8). (C) The pre‐miRNA is transported to the cytoplasm through the exportin 5/RanGTP complex. (D) The GYM motif is recognized by the dsRBD (residues R1855 and E1859) and RNase III (RIIID) domains of DICER to allow pre‐miRNA binding and indicate cleavage sites. (E) In the dicing state, the DUF283 and N‐terminal helicase regions become flexible, the C‐terminal of dsRBD is moved away from the catalytic center, and the 5′ and 3′ ends of the pre‐miRNA are fixed in the 5′ and 3′ pockets, respectively. Then the pre‐miRNA is processed into the miRNA duplex by DICER. (F) The miRNA duplex is unchained by the argonaute proteins (AGO) as well as the RNA helicase complex to generate the mature miRNA.

In summary, these two works complement each other by investigating both the GYM motif of pre‐miRNA and the structure of hDICER–pre‐miRNA^GYM^ in the dicing state, illustrating the mechanism of how DICER specifically recognizes and efficiently processes pre‐miRNA. The discovery of the GYM motif has provided a new idea and direction for future studies on the mechanism of gene substrate recognition by similar enzymes, which is a landmark. Meanwhile, we are no longer constrained by our inability to capture the catalytic state thanks to the discovery of the structure of hDICER–pre‐miRNA^GYM^ at the dicing stage.

In order to facilitate future clinical translation of studies on the mechanism of DICER specific recognition and processing of pre‐miRNA, researchers can explore the relationship between the effects of different GYM motifs on DICER processing of pre‐miRNA and the occurrence of miRNA‐associated diseases, laying a solid foundation for the development of future RNA therapies for clinical miRNA‐associated diseases. In addition, based on the new discovery of important structural regions of DICER in this study, novel small molecule drugs targeting DICER can be designed and developed to better regulate the process of DICER processing pre‐miRNA, thereby achieving regulation of miRNA expression levels. In conclusion, the research on the molecular mechanism of efficient recognition and processing of pre‐miRNA by DICER has significant application prospects in basic research and the development of human disease treatment and intervention methods.

## AUTHOR CONTRIBUTIONS

Quan Zheng conceived the manuscript. Qi Weng and Quan Zheng wrote the manuscript. Qi Weng prepared the figure. Qi Wu revised and proofread the manuscript. All authors have read and approved the article.

## CONFLICT OF INTEREST STATEMENT

The authors declare they have no conflicts of interest.

## ETHICS STATEMENT

Not applicable.

## Data Availability

Not applicable.
